# Are remote health clinics primary health care focused? Validation of the Primary Health Care Engagement (PHCE) Scale for the Australian remote primary health care setting

**DOI:** 10.1017/S1463423623000592

**Published:** 2024-01-05

**Authors:** Kylie McCullough, Gemma Doleman, Melissa Dunham, Lisa Whitehead, Davina Porock

**Affiliations:** School of Nursing and Midwifery, Edith Cowan University, Joondalup, Perth, Western Australia

**Keywords:** instrument development, measurement, nurses, nurse practitioners, primary health care, remote communities, survey

## Abstract

**Aim:**

To test and validate a measure of primary health care (PHC) engagement in the Australian remote health context.

**Background:**

PHC principles include quality improvement, community participation and orientation of health care, patient-centred continuity of care, accessibility, and interdisciplinary collaboration. Measuring the alignment of services with the principles of PHC provides a method of evaluating the quality of care in community settings.

**Methods:**

A two-stage design of initial content and face validity evaluation by a panel of experts and then pilot-testing the instrument via survey methods was conducted. Twelve experts from clinical, education, management and research roles within the remote health setting evaluated each item in the original instrument. Panel members evaluated the representativeness and clarity of each item for face and content validity. Qualitative responses were also collected and included suggestions for changes to item wording. The modified tool was pilot-tested with 47 remote area nurses. Internal consistency reliability of the Australian Primary Health Care Engagement scale was evaluated using Cronbach’s alpha. Construct validity of the Australian scale was evaluated using exploratory factor analysis and principal component analysis.

**Findings:**

Modifications to suit the Australian context were made to 8 of the 28 original items. This modified instrument was pilot-tested with 47 complete responses. Overall, the scale showed high internal consistency reliability. The subscale constructs ‘Quality improvement’, ‘Accessibility-availability’ and ‘population orientation’ showed low levels of internal consistency reliability. However, the mean inter-item correlation was 0.31, 0.26 and 0.31, respectively, which are in the recommended range of 0.15 to 0.50 and indicate that the items are correlated and are measuring the same construct. The Australian PHCE scale is recommended as a tool for the evaluation of health services. Further testing on a larger sample may provide clarity over some items which may be open to interpretation.

## Introduction

Primary health care (PHC) services are fundamental to any health system as they provide a range of ‘first line’ urgent care medical services as well as health promotion and disease prevention, public health, rehabilitation and palliative care, as close as practicable to the communities they serve (Prade et al., 2023). Operationalising this vision for PHC requires investments in research and monitoring for quality and safety and evaluating changes and improvements in care (World Health Organisation, 2020).

In Australia, approximately 85% of the continent is considered ‘remote’ or ‘very remote’ (Australian Bureau of Statistics, 2016). These remote communities service mining, tourism, agriculture and First Nations (Aboriginal and Torres Strait Islander) communities. These remote populations are small and geographically isolated, and residents experience poorer health outcomes than other Australians. Health service provision in these communities usually consists of a nurse-led clinic which provides acute care and emergency health response as well as a range of health promotion, public health and social health activities (McCullough et al., 2020). Many clinics are run by local Aboriginal and Torres Strait Islander organisations, others run by government departments, and some clinics are run by other private organisations. The range of available services varies between communities. Some clinics have resident General Practitioners, Aboriginal Health Practitioners and allied health services, and most provide specialist services via visiting teams or telehealth.

Remote clinics (not hospitals with inpatient services), therefore, aim to provide a comprehensive service that meets the community’s health needs within practical and financial limitations (McCullough et al., 2020). However, the complex nature of remote health service provision has not been well studied. In particular, the degree to which health services meet the expectations of a comprehensive PHC service is not known. A review of the literature (McCullough et al., 2023) identified a psychometrically validated tool to measure health service engagement with PHC philosophy as a measure of quality care in rural and remote PHC settings in Canada (Kosteniuk et al., 2017; Kosteniuk et al., 2016). This paper reports on a study to validate this tool for the Australian remote setting and explore the similarities and differences between remote health services as a baseline measure to track progress in health care reform.

## Background

The National PHC Strategic Framework, endorsed by the Australian government at state and federal level, presents an approach for a stronger PHC system in Australia and highlights the importance of PHC services (Standing Council on Health, 2013). The framework outlines the vision and strategic outcomes for health care reform and was developed in response to evidence that showed that ‘… health systems with strong PHC are more efficient, have lower rates of hospitalisation, fewer health inequalities and better health outcomes including lower mortality’ (Standing Council on Health, 2013: p.v).

However, to articulate and evaluate the impact of health services reform towards a PHC model, it is necessary to understand how PHC principles affect the practice of those delivering the care. To that end, it is not known how reorientation of health systems towards a PHC model has affected nursing practice or what impact a PHC-focused nursing workforce has on health outcomes. Furthermore, Bourke et al. (2013) discuss a lack of understanding of how concepts such as PHC and the social determinants of health are ‘…theorised, applied and operationalised in rural and remote health policy, practice and research’ (p.66). Besner (2004) suggests that further research investigating nurses’ conceptualisations of PHC and how it shapes their practice is needed. Considering the perspectives of nurses on how their health services are implementing PHC into their practice may also lead to refinement and improvements in PHC objectives, service models and workload measures.

A review of measures of nursing care in PHC settings determined the implementation of PHC principles as a measurable element of quality care (McCullough et al., 2023). The Primary Health Care Engagement (PHCE) scale (Kosteniuk et al., 2017; Kosteniuk et al., 2016) was identified as an instrument to explore within the Australian context. The PHCE scale was developed in Canada from a literature review, expert consultation and pilot testing (Kosteniuk et al., 2016) with registered nurses (RNs) working in rural and remote areas. This preliminary scale was then included in a larger study of 1587 participants and the psychometric properties established (Kosteniuk et al., 2017). The 28-item scale measures key elements of PHC: accessibility and availability of health services, community participation and intersectoral teamwork, interdisciplinary collaboration, person-centred care, continuity of care, population characteristics and quality improvement activities (Kosteniuk et al., 2017). The PHCE scale was shown to have good internal consistency reliability and construct validity. Only one instance of use of the PHCE scale other than instrument development could be found in the published literature and that was part of a mixed-methods study to evaluate the work of nurse practitioners (NPs) in rural regions of Canada (Wilson et al., 2021). Further validation of the scale is required (Kosteniuk et al., 2017). Validation of the PHCE scale for the Australian context will provide a new mode of evaluation of health services in remote areas.

Understanding the nurse’s perspective could contribute to a deeper understanding of the relationship between nursing practice and health outcomes within the remote setting. Further research which measures the difference (if any) in health outcomes for communities in relation to the degree of PHC delivery would aid policy makers and employers in their decisions about the resources needed in remote communities.

### Aims and objectives

This study aims to measure one aspect of quality care within the remote PHC setting by evaluating nurses’ perspective of the alignment of the health service in which they work with PHC principles.

This study has three main objectives:

1. To assess the face and content validity of the Canadian PHCE scale in the Australian remote setting by a panel of expert remote area nurses and construct the Australian version of the PHCE ready for testing.
2. To assess reliability and construct validity of the Australian PHCE with a sample of practicing remote area nurses.
3. To quantify remote area nurses’ perception of the degree to which their health services are engaged with key principles of PHC using the Australian PHCE.
  - To determine if age, work experience, size of service or ownership of service (government or First Nations) has any impact on the nurses’ perception of engagement with PHC.

## Methods

### Stage one: instrument face and content validity

In stage one, nurses with expertise in rural and remote nursing were invited to assess content and face validity of the PHCE scale developed by Kosteniuk et al. (2017). Focus group participants were drawn from an existing remote research collaboration between Edith Cowan University, James Cook University, Flinders University and CRANAplus (remote area nursing’s national representative body). Members were invited to participate and to nominate additional remote area nurses known to them.

A panel of 12 members was established with expertise in rural and remote health. A validation questionnaire was developed in Qualtrics using a series of demographic questions and the original 28-question PHCE scale. Respondents were asked to assess each question for representativeness (how well the question represents the Australian remote context) and clarity (how easy it is to understand and respond to the question). A text box was provided for respondents to comment and make suggestions.

Responses were collated and rated as a percentage against representativeness and clarity for each question. All comments by each panel member were also tabulated. A meeting between the research team was conducted to examine and discuss expert panel responses. Any response that rated 80% or lower was specifically addressed through discussion between the research team. However, three questions were sent back to the expert panel for comment. Responses were received, and the authors of original scale were contacted for clarification where needed.

### Stage two: pilot-testing the instrument

In stage two, the modified scale was pilot-tested with remote area nurses. PHCE scale items were answered on a five-point Likert scale of strongly agree, somewhat agree, neither agree or disagree, somewhat disagree and strongly disagree. Five items were negatively worded identically to Kosteniuk’s original. Item 25 was a negatively worded in Kosteniuk’s work, but after consultation with the panel of experts, it was reworded for this study and became positively worded. This will be explained in further detail below. These were reverse-coded before a total score was calculated representing engagement with PHC principles in the workplace. The total score was used to test hypotheses.

Quantitative data were exported into SPSS version 28 (IBM Corp, 2019) and assessed for normality and/or transformed where appropriate. Demographic data were explored utilising descriptive statistics, including frequencies, mean, standard deviation and range. Where surveys were missing 25% or less of items, case mean imputation was performed to allow inclusion of participant data. Surveys missing more than 25% of items were discarded, in line with analysis by Kosteniuk et al. (2016).

The PHCE scale was re-administered to participants via email link within a 2–4-week period from first completion. People who completed the survey twice were offered to go in a draw to win one of five gift cards valued at $100 which was donated by CRANAplus.

Consistency of the PHCE scale in an Australian context was evaluated using Cronbach’s alpha (Cronbach, 1951), which measures correlation of each item with one another, by comparing the variance of sum of the independent variables with the sum of the variances for each variable. The higher the correlation, the higher the consistency. A value of > 0.7 was considered to show a high degree of consistency. Construct validity of the PHCE scale was evaluated using exploratory factor analysis, using principal component analysis following the methods described by Kosteniuk et al. (2017).

#### Recruitment of participants

There are approximately 1000 RNs working in remote community health centres (those without inpatient facilities) across Australia. Participants were eligible to complete the survey if they were currently or had worked in a remote health service (without inpatient services) within the last six months.

The survey link was distributed via the weekly CRANAplus email to members and via Facebook groups related to remote area nursing. Participants were encouraged to share the survey link among their networks. This resulted in a simple cross-sectional sample over the period September 2021–October 2022.

### Stage one results

A panel of 12 members was established with expertise in rural and remote health (Table 1). Questionnaires were sent out to 12 experts between September and November 2020. Ten expert’s responses were received and analysed.

**Table 1.** Participant demographic characteristics

Age Mean (SD)		Female *n* (%)		Male *n* (%)	
52.68 (9.69)		43 (92)		3 (6)	
Qualification *n* (%)					
Diploma	Bachelor	Graduate certificate	Graduate diploma	Masters	
1 (2.1)	10 (21)	16 (34)	6 (12.8)	14 (30)	
Registration *n* (%)					
Registered nurse	Registered midwife	Registered nurse/midwife	Nurse practitioner	Registered nurse/registered midwife/nurse practitioner	
35 (80)	2 (4)	7 (15)	1 (2)	2 (4)	
Years in rural and remote health *n* (%)					
0–5 years	6–10 years	11–20 years	21–30 years	31–40 years	
12 (26)	10 (21)	19 (40)	5 (11)	1 (2)	
Years in role *n* (%)					
0–5 years	6–10 years	11–20 years	21–30 years	31–40 years	Missing
27 (57)	10 (21)	2 (4)	1 (2)	1 (2)	7 (15)
Organisational structure of current most recent health service *n* (%)					
Government	Aboriginal community-controlled	Not-for-profit			
23 (49)	23 (49)	1 (2)			
Size of town *n* (%)					
100–300	301+	Missing			
11 (23)	31 (67)	5 (11)			
Residence *n* (%)					
I would probably live here even if I wasn’t working at the health service					5 (11)
I generally stay in town on days off and this community is my current home					14 (30)
I stay in the community for my shifts and usually travel elsewhere for my days off					7 (15)
I work on short contracts (less than three months) in communities					11 (23)
I work for an agency					3 (6)
Participants live in one small community and drive to another small community for work					7 (15)

#### Panel demographics

The average age of the 10 experts was 48, and all were female. One member identified as an Aboriginal and/or Torres Strait Islander person. All were RNs with one also being a NP, two also being dual-registered midwives, and one being an RN, NP and RM. The average number of years working as an RN was 25 (range 14–40 years), average as an NP 6 years (range 3–10) and average number of years working in remote health was 17 (range 8–42 years). All bar one held postgraduate qualifications, and the panel represented a wide range of roles from predominantly clinical (*n* = 5), education and management (*n* = 3) and combined clinical, research and management roles (*n* = 4). Seven panel members were based in the Northern Territory, three in Queensland, one in Western Australia and one in Tasmania.

#### Panel responses

In addition to reviewing the scale items for content validity, the expert panel was asked to comment on the survey demographic questions proposed by the research team. As researchers, we aimed to describe the sample in terms of their age, gender and ethnicity and professional experience. We also wanted to collect community demographic data because we hypothesised that nurse’s perspectives on the level of engagement with PHC philosophy may be related to their professional experience in remote areas, the community characteristics and employer type. The demographic questions can be found in appendix A: PHCE scale – Australian version.

After the first round of responses from the expert panel, 14 items (1, 2, 7, 9, 10, 11, 16, 19–21, 25–28) from the PHCE scale scored less than 80% in either or both the representiveness and clarity section. Items 7, 9 and 26 were not changed as the research team considered the comments were clarified by changes made in other items. Items 19, 20 and 21 required minor changes to the terminology used for health care workers in Australia to Aboriginal Health Workers, nurses, General Practitioners, allied health and visiting specialists.

Item 11 received a response of 100% representativeness and 80% for clarity; however, the research team identified that the assumption with the original item was that people make appointments, which is not relevant in remote communities in the Australian context. Other items that received above 80% from expert panel were still considered by the research team. Minor variations to items were undertaken such as capitalising words for clarification. Throughout the scale items, the wording of ‘My workplace’ was changed to ‘My current or most recent workplace’ to capture responses to a specific workplace and not to workplaces in general.

After reviewing the responses, three items remained problematic 10, 11, and 25 and were sent back to the 10 expert panel members for their guidance and suggested changes or comments. A total of seven second round responses were received towards the end of May 2021 from the expert panel. A second review meeting of the research team was held on 15 June 2021, and all items for the Australian version of the PHCE were finalised through consideration of expert panel feedback and discussion amongst researchers.

### Stage two results: the pilot study

#### Sample characteristics

A total of 47 completed surveys were returned and fully completed via online tool Qualtrics. The potential pool of respondents were 686 remote area nurses and 23 NPs who were members of CRANAplus (personal communication, 22/03/2022). Most of the participants were female (92%) and over the age of 51 years (61%). One participant identified as an Australian Aboriginal person. Seventy-seven per cent (*n* = 36) had a postgraduate degree. Seven participants had dual-registered nurse/registered midwife registration. Three participants were NPs. Thirty-five (74%) participants had been working in the rural and remote setting for six years or longer. Twenty-seven participants (57%) had worked in their current role for less than five years. Twenty-three (49%) participants worked in Aboriginal Community Controlled Health Services and 23 (49%) worked in government-controlled health services. Sixty-seven per cent (*n* = 31) of participants work in a community with more than 300 people. See Table 1 for detailed participant demographics.

#### Reliability testing

Internal consistency of the Australian PHCE Scale was explored. The Cronbach’s alpha (α) was calculated to allow for examination of the internal consistency of the scale and subscale constructs and a comparison of scores (Table 2) with the original scale and subscales created by Kosteniuk et al. (2017). Overall, the survey showed high internal reliability and consistency. The subscale constructs ‘Quality improvement’, ‘Accessibility-availability’ and ‘population orientation’ showed low levels of internal consistency and reliability. However, the mean inter-item correlation was 0.31, 0.26 and 0.31, respectively, which are in the recommended range of 0.15 to 0.50 and indicate that the items are correlated and are measuring the same construct. The psychometric testing suggests that the items in the ‘Quality improvement’ and ‘Accessibility-availability’ subscale be revisited for relevance to the Australian context and tested with a larger sample.

**Table 2.** Cronbach alpha (α)

	This study	Kosteniuk et al study
Overall scale	.918	.89
Subscale		
Quality improvement (1, 2)	.475	.88
Community participation (3, 4, 5, 6)	.844	.86
Patient-centred care (7, 8, 9, 10)	.835	.83
Accessibility-availability (11, 12, 13, 14)	.579	.80
Intersectoral team (15, 16, 17, 18)	.863	.78
Interdisciplinary collaboration (19, 20, 21)	.834	.77
Continuity (22, 23, 24)	.841	.61
Population orientation (25, 26, 27, 28)	.641	.61

In Table 2, the three subscales, ‘Quality Improvement’ and ‘Accessibility-availability’ showed poor internal consistency, contrary to Kosteniuk’s study. The difference in Cronbach’s alpha for these three subscales when compared to Kosteniuk’s study was surprising given the similarity between the two instruments in the other subscales. The question is how, by adding examples of common indicators to clarify the concept, this would result in poorer internal consistency? If these examples were added to the original version and tested again in the Canadian context would that change the internal consistency? Or is it that the items are not as reliable in the Australian context? We think that the Quality improvement subscale may be influenced by our addition of examples of common indicators and measures of quality data which clarified the meaning of the item. As our expert panel found a lack of clarity of the items in these subscales without examples, this suggests further testing in both Canada and Australia would be beneficial.

Furthermore, the Accessibility-availability subscale may not accurately measure the complex construct of ‘access’ to care (Levesque et al., 2013) within the Australian setting because health services are theoretically available to all patients at all times, as nurses in remote areas are on-call for 24 h. This may be different to the Canadian context.

However, while ‘Population orientation’ indicated poor internal consistency, the results of this study indicated a slightly higher reliability score than that of Kosteniuk’s study. This may be attributed by the change in wording of item 25 from a negatively worded to positively worded item in this present study helping participants understand the item better. As mentioned above, this difference may be due to the items not being relevant to the Australian context. These domains should be reviewed in studies with larger sample size.

The Australian PHCE scale aims to determine the degree to which a service reflects engagement with PHC principles as determined by practicing remote area nurses in Australia. The total score for each participant was calculated to identify the overall level of engagement. A numerical score of 5 was attributed to the response Strongly Agree and a score of 1 was attributed to Strongly Disagree for each item of the new scale with a potential score between 28 and 140. Care was taken to reverse negatively worded items before summing the scores. Descriptive statistics demonstrated a range of responses from participants indicating their perception of engagement with PHC principles in their practice. The mean perceived engagement score was relatively high for this study at 104.3, see Table 3 for central tendency and dispersion metrics.

**Table 3.** Central tendency and dispersion metrics for the total engagement scores

Mean	104.3
Mode	108
Median	106
Range (min-max)	55–135
Standard deviation	18.28

Bearing in mind the small sample size and skewed distribution, non-parametric tests were performed to determine if any of the demographic items could explain the variation in engagement scores. Age, experience, size of community or being at a government or non-government clinic showed no significant difference using non-parametric tests. To investigate further, the PCHE scale scores were separated into a low-, middle- and high-level engagement group. Responses were then compared with demographic variables to explore engagement levels. As Table 4 shows, this categorisation does not reveal potential reasons for the variation in engagement score based on demographic characteristics except for experience in current role where descriptively there is lower engagement with less experience in current role. However, using Kruskal–Wallis H-test experience in current role was not statistically significant on level of engagement X2 (2) = 1.64, *P* = 0.440. The distributions of the experience in current role were not similar for all groups, as assessed by visual inspection of a boxplot. Although it would be useful to revisit, this in a larger sample.

**Table 4.** Demographic comparisons for low, middle and high level of engagement

Demographic	Low level of engagement (*n* = 17)	Middle level of engagement (*n* = 15)	High level of engagement (*n* = 15)
Score range	55–100	101–116	117–135
Age average	49.3	54.3	54.9
Gender			
Male	1		2
Female	15	15	13
Highest qualification			
Diploma		1	
Bachelor	5	2	3
Graduate certificate	5	4	7
Graduate diploma	1	5	
Masters	6	3	5
Role			
RN	14	9	12
RM		1	1
RN/RM	3	3	1
Nurse practitioner			1
RM/RN/Nurse practitioner		2	
Experience in rural and remote health care in months			
	166 (range 24–485)	127.4 (range 50.25–240)	152.06 (range 12–318)
Experience in current role in months			
	31.07 (range 1 week–182 months)	51.48 (range 2 weeks–168.75 months)	51.0 (range 0–252 months)
Organisational structure of current most recent health service			
Government	10	5	8
Non-government	6	10	7
Not-for-profit	1		
Size of town			
Size 0–300	3	4	4
301+	11	11	9
Missing	3	0	2

The Mann–Whitney U test was used in this study to compare the difference in the response means with PHC engagement between the two independent employment groups, non-government and government. There was no statistically significant difference in responses between the two employment groups.

For test retest reliability, participants were invited to repeat the survey two weeks after completing the initial survey. Unfortunately, only five responses could be matched, so statistical testing was not possible. The total engagement score between the five respondents showed stability in responses overtime. See Table 5.

**Table 5.** Test–retest reliability scores

Participant	Initial scale distribution total engagement scores	Follow-up scale distribution total engagement scores
1	55	73
2	108	102
3	101	108
4	121	117
5	120	123

## Discussion

This study aimed to evaluate an existing instrument designed to measure the extent to which health services are consistent with PHC principles. Minor modifications were recommended to reflect the Australian context, and the Australian version of the PHCE scale supports health service evaluation consistent with expectations of the National Safety and Quality Primary Health Care standards (Australian Commission on Safety and Quality in Health Care, 2020). Of particular value is that this instrument elicits the perspective of nurses who are the primary care providers within PHC settings, for whom there are few existing measures that relate directly to their care provision (McCullough et al., 2023). That said, we encourage the uptake and use of the scale by other health providers, such as Aboriginal Health Practitioners and General Medical Practitioners.

Ensuring that health services respond to community need is a fundamental principle of PHC. To that end, Aboriginal Community Controlled Health Services are well positioned to orientate their services towards programmes focused on the social determinants of health and community health priorities because they are governed by the community (Pearson et al., 2020). However, we did not find differences between Government and Aboriginal Community Controlled Health Service providers. This was interesting because we expected higher overall scores from Aboriginal Community Controlled Health Organisations, with their emphasis on comprehensive PHC, community engagement and health promotion (National Aboriginal Community Controlled Health Organisation, 2021). It is possible that the instrument is not effectively measuring factors that are significant for Aboriginal and Torres Strait Islander peoples and health service organisations such as incorporating ‘…spirit, land, environment…’ in care as can be seen in the National Aboriginal Community Controlled Health Organisation (NACCHO) PHC definition:

PHC is a holistic approach which incorporates body, mind, spirit, land, environment, custom and socio-economic status. PHC is an Aboriginal construct that includes essential, integrated care based upon practical, scientifically sound and socially acceptable procedures and technology made accessible to Communities as close as possible to where they live through their full participation in the spirit of self-reliance and self-determination. The provision of this calibre of health care requires an intimate knowledge of the community and its health problems, with the community itself providing the most effective and appropriate ways to address its main health problems, including promotive, preventative, curative and rehabilitative services (National Aboriginal Community Controlled Health Organisation, 2009 p. 6).

Further research that specifically seeks to explore this perspective by including participants who identify as Aboriginal and/or Torres Strait Islander people and/or includes specific measures, such as an instrument to measure cultural capability of the workforce, may assist in further development of this instrument for the Australian context (West et al. (2018). This is of particular importance as cultural safety principles state that it is the recipient of care that determines the safety of the service (Curtis et al., 2019). Furthermore, two items (10 and 25) deserve special mention because they generated the most discussion amongst the expert panel and the areas of modification in relation to cultural safety principles. Item 10: *My workplace is a safe place for patients to receive health care services* was considered too broad as ‘safety’ could refer to cultural, clinical or personal safety. We contacted the original authors and clarified that the intent was a measure of cultural safety, as access to care is impacted if patients do not feel that the health service is acceptable. However, in clarifying these items, we realised that feelings of physical safety and confidence in the clinical safety were also important enablers for consumers to access care. In addition, awareness of services provided, the ability and consumers capacity to seek care in terms of power also impact on access to health care services. This demonstrates that access to care is complex construct that is broader than simply having a service available (Levesque et al., 2013). Furthermore, the disparity in Cronbach’s measure demonstrated in Table 2 may also be a result in differences in interpretations of access and availability. Therefore, further development of these items to include other dimensions of safety such as cultural safety, physical safety within the clinic for staff and patients or patient confidence in the clinical safety of the service along with a broader understanding of the consumers barriers to accessing the services would be useful.

Item 25 *My workplace is slow to respond to the health needs of the community* deserves mention because the expert panel felt that health services were often well prepared and responsive to acute clinical health needs – as seen in the COVID-19 pandemic and vaccine roll-out (Fitts et al., 2020) but were slow or ineffectual in responding to social or chronic health needs (McCullough et al., 2021). The panel questioned whether this item was about the speed of the response, or the effort/resources invested in addressing the problem. Interestingly, this item originated from Dahrouge et al. (2009) item ‘Are you able to change health care services or programs in response to specific health problems in the community?’. We interpreted this item as emphasising the *response* of health care services to social determinants of health and health literacy rather than the *speed* of response. We justified this as an indication of health service awareness and intention to address broader issues raised by the community, rather than an immediate response to a clinical condition, as favoured by Aboriginal Community Controlled Health Services (Pearson et al., 2020). Our change to this item shifted this item from a negatively worded item to a positively worded item making comparisons less straightforward.

Whilst the focus of this study was on instrument validation, we found that the mean engagement score of 104 indicates a generally positive engagement with PHC principles. This finding is supported in other qualitative studies which describes providing PHC as the aim of nursing care and the resultant job satisfaction as a factor in retention and quality of care (McCullough et al., 2020). Monitoring engagement over time may be a useful indicator of the effectiveness of initiatives aimed at improving recruitment and retention of staff in these challenging areas.

Of note for future research is that the total score generated from this scale assumes that all items are of equal importance. We suggest that researchers explore the items to identify those which are nurse-specific (as opposed to systemic) and therefore may be a measure of nursing quality of care. Identification of items that measure systemic variables such as skill mix and availability of staff may further increase the utility of this instrument.

### Limitations

Whilst we included participants who worked within Aboriginal Community Controlled Health Services, a limitation of this study was the lack of Aboriginal and/or Torres Strait Islander participants, so we cannot be sure that this instrument is measuring concepts of interest to these groups who make up a large proportion of residents of remote communities. Indeed, as this measure is from the perspective of nurses, more research is needed from the consumer perspective in order to demonstrate more valid findings.

Unfortunately, the low response rate to this survey and in particular the test–retest group limits the ability for us to report on variance. Larger sample sizes would increase the ability to understand the factors that contribute to effective PHC services and identify areas for improvement. Further, whilst the total scores between the test–retest groups were similar, and thus reliability can be assumed, the small sample size means this should be treated with caution until further research is conducted.

The low response rate in this study was disappointing. Other authors have described reasons for low responses that include rising rates of refusal, in part due to an increase in the number of surveys and thus becoming fatigued (Karlberg, 2015). With the increase in smartphone and online survey technology, administering surveys have become low cost and easy to distribute (O’Reilly-Shah, 2017; Field, 2020). When the COVID-19 pandemic hit in March 2020, online surveys became the only mechanism for many researchers to continue their work and have been shown that the increase has led to survey fatigue and reduced response rates (Field, 2020; De Koning et al., 2021). Furthermore, the COVID-19 pandemic decreased the availability and capacity of nurses working in remote areas which may have had an impact on their willingness to participate in research.

Strategies such as keeping the questionnaires brief, using simple and precise language, providing a personalised invitation, translating the survey into relevant languages, setting deadlines for responses, providing financial incentives or explaining how the participant will benefit from completing the survey and regular reminders are suggestions for increasing response rates (Pecoraro, 2012; De Koning et al., 2021). These strategies could be implemented in future studies to achieve a higher response rate.

### Implications for practice and research

Strong PHC systems impact positively on health and deliver substantial cost savings, to that end, the Australian government PHC 10-year plan includes strategies to focus on the nursing workforce and takes steps to improve evaluation and research of PHC services (Australian Government Department of Health, 2022). Furthermore, the World Health Organization operational framework is devoid of instruments that measure overall adherence of a service to PHC principles (World Health Organisation, 2020). This instrument may assist in reaching these goals.

### Conclusion

Despite some limitations, this study resulted in the modification of an existing measurement tool for the Australian context. Employers, communities and researchers may find that this provides a new method of health service evaluation which contributes to the monitoring and understanding of quality care in PHC settings. Further research with larger sample sizes and within different settings, such as Aboriginal Community Controlled Health Services, would improve the reliability and validity of this measure.
